# Improving Deproteinization in Colombian Latex from *Hevea brasiliensis*: A Bibliometric Approximation

**DOI:** 10.3390/polym14194248

**Published:** 2022-10-10

**Authors:** Fabian Hernandez-Tenorio, Héctor Arroyave-Miranda, Alejandra M. Miranda, Sandra M. González, Carlos A. Rodríguez, Alex A. Sáez

**Affiliations:** 1 School of Applied Sciences and Engineering, Universidad EAFIT, Medellín 050022, Colombia; 2 Biological Sciences and Bioprocesses Group, School of Applied Sciences and Engineering, Universidad de EAFIT, Medellín 050022, Colombia; 3 Engineering, Energy, Exergy and Sustainability Group (IEXS), School of Applied Sciences and Engineering, Universidad EAFIT, Medellín 050022, Colombia

**Keywords:** natural rubber, protein, deproteinization, bibliometric analysis, latex

## Abstract

Natural Rubber Field Latex (NRFL) allergens restrict its use in some markets due to health-threatening allergic reactions. These molecules are proteins that are related to asymptomatic sensitization and hypersensitivity mediated by immunoglobulin E (IgE). Although NRFL allergens have been investigated since the 1980s, there are still gaps in knowledge regarding the development of deproteinized natural rubber (DPNR). Therefore, in this study, the deproteinization of NRFL from the lower basin of the Cauca River, Antioquia-Colombia was evaluated using eight systems. The highest removal value was 84.4% and was obtained from the treatment containing SDS (Sodium dodecyl sulfate), Urea, and Ethanol. It was also possible to determine that at high concentrations of SDS, removal percentages higher than 70% are reached. On the other hand, all deproteinizing systems decreased NRFL Zeta potentials without self-coagulation, suggesting enhanced colloidal stability in DPNR latex. On the other hand, the bibliometric analysis presented technological advances in DPRN through different parameters and bibliometric networks. The analysis presented makes an important contribution from the bibliometric approach that could be positive for the development of research on DPNR.

## 1. Introduction

Natural rubber (NR) is an important biopolymer in the development of different applications because it is used in more than 50,000 pharmaceutical, cosmetic, and industrial products, among others [1,2,3,4,5]. NR possesses valuable properties such as elasticity, resistance to abrasion, impact and tearing, efficient heat dispersion, and malleability at low temperatures; therefore, it cannot be completely replaced by synthetic rubber [6]. 1,4-polystyrene is the compound that constitutes the NR and it is processed from field latex (FL) [7], whose only commercial source is *Hevea brasiliensis* trees [8]. The world production of NR is mainly in Asian countries such as Indonesia and Thailand with 3.30 and 4.85 million metric tons obtained in 2019, respectively. While the Amazon region originates the *H*. *brasiliensis* species, it only generates 2% of world production [9].

The NR currently presents two problems, one of them is the increase in demand that causes insecurity in biopolymer supplies [10]. Additionally, high labor costs, price instability, *H. brasiliensis* diseases, and the prohibition of deforestation generate interest in the search for alternative NR-producing plants such as *Taraxacum kok-saghyz* and guayule *Parthenium argenta-tum,* among others [9,11]. On the other hand, the allergens of NR latexes restrict their use in some markets due to allergic reactions that put health at risk [12].

NRFL allergens are proteins that are associated with asymptomatic sensitization and hypersensitivity mediated by immunoglobulin E (IgE) [13]. According to the Subcommittee on Allergen Nomenclature of the World Health Organization (WHO)/International Union of Immunological Societies (IUIS), 15 protein molecules known as Hevea allergens (Hev b1 to Hev b15) have been registered; however, additional allergens that may exacerbate health problems due to allergic reactions continue to be investigated [14]. Allergens present clinical manifestations due to type I hypersensitivity reactions and vary according to exposure, whether percutaneous, parenteral, mucosal, or cutaneous. Skin affections due to allergens include immunological contact urticaria, which is the most common manifestation of a latex allergy. Other affections are DCP contact dermatitis and allergic contact dermatitis (hypersensitivity type IV), the latter is due to the additives used in the processing of NRFL [15]. Respiratory problems such as asthma, cough, conjunctivitis, rhinitis, and eosinophilic bronchitis are also known to originate from contact with latex particles [14,16].

NR is present in latex as rubber particles ranging from 0.2 to 4.0 μm in average diameter [17]. These are surrounded by a layer of components that are considered non-rubber. The surface layer of rubber particles contains some enzymes and rubber transferases, which are associated with the biosynthesis of rubber, as a kind of membrane protein. Thus, these proteins can be expected to adhere to the surface of the rubber particles by the excluded volume effect of the lipids. In addition, other proteins, such as pathogenic proteins or allergens, are strongly attached to the surface of the rubber particles [18]. Therefore, the deproteinization or removal of proteins from the NR must be carried out at the latex stage, since almost all the proteins present in the NR are found on the surface of the rubber particle through chemical or physical interactions [19]. The main deproteinization methods are based on the denaturation of proteins by means of agents such as urea, proteolytic enzymes, surfactants, and alkali. For example, Urea is effectively used in the presence of a surfactant, while proteolytic enzymes hydrolyze proteins into small peptides; however, a long incubation time and strict temperature control are necessary in this method. On the other hand, washing NRFL with ionic surfactants such as SDS and non-ionic a polyethylene glycol effectively reduce the proteins in the rubber particles by transferring them to the serum phase. Alkalis such as sodium hydroxide (NaOH) also act as denaturants to hydrolyze proteins [20].

Although NRFL allergens have been investigated since the 1980s, there are still gaps in knowledge regarding the development of DPNR. Additionally, the potential risk of protein allergies in the use of NR products for some sensitized users has created a continuing demand for NRPD [10,12]. Therefore, the objective of this article was to evaluate different deproteinizing agents of NRFL from the lower basin of the Cauca River, Antioquia-Colombia with the purpose of adding value to the NR produced by cooperatives of *Hevea brasiliensis* farmers in the area that faces major socio-economic problems.

A comprehensive review of technological advances for NR deproteinization is shown. In addition, this review focuses on providing a bibliometric analysis of DPNR, in which trends in deproteinization methods, effects of deproteinization on NR properties, scientific production, and applications of DPNR are indicated. The presented analysis makes an important contribution from the bibliometric approach that could be positive for the development of research on DPNR and provides some suggestions to researchers working on the subject.

## 2. Bibliometric Analysis of Deproteinized Natural Rubber

### 2.1. Scientific Production

Currently, the problem of allergies caused by some proteins present in latex is well documented [21,22,23,24]. However, there are gaps in the knowledge that link the effects that deproteinization methods may have on the properties and quality of natural rubber. For this reason, it is pertinent to evaluate through scientometric tools the technological advances related to deproteinized natural rubber. Consequently, a systematic search was carried out in the Scopus scientific database under search criteria established by means of the equation TITLE-ABS-KEY (“natural rubber” AND (“protein” OR “deproteinized” OR “DPNR”)). AND (LI-MIT-TO (DOCTYPE, “ar”) OR LIMIT-TO (DOCTYPE, “re”)). The bibliometric parameters, total number of citations, average number of citations per article, and categorization of publications with the most citations were calculated from the Bibiometrix package of R commander (x64. 4.1.0). The VOSviewer version 1.6.16 and Cor-Text Manager software types were used to determine bibliometric networks such as Co-occurrence maps, Co-authorship, and contingency matrix. It is noteworthy that the compiled documents were filtered in order to avoid the repetition of terms with abbreviations and hyphens [25,26].

**Figure 1:**
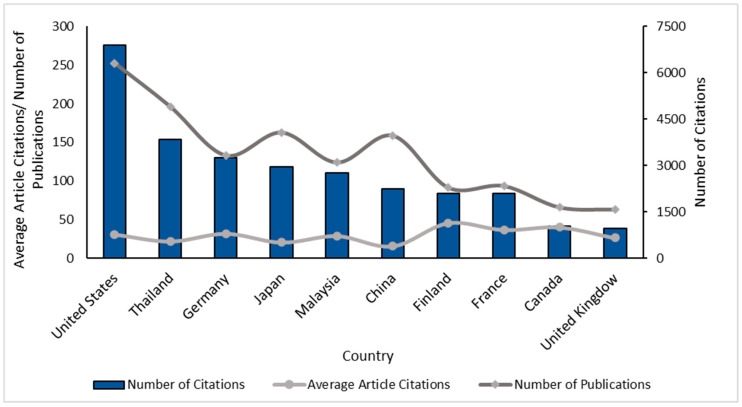
Leading countries in publications on DPNR, data-based Scopus reports.

The relevance of published research on deproteinized natural rubber was analyzed using scientometric tools; consequently, Figure 1 shows the results on the categorization of the leading countries in scientific production in DPNR, finding the United States with the highest number of citations (6888), followed by Thailand (3853). Likewise, Finland and Canada were identified with the highest average number of citations per article with 45.76 and 40.26, respectively, which is why the importance of these publications in the area of study and their possible use as references of interest for other investigations is evident.

On the other hand, the Bibliometrix tool made it possible to classify the publications on deproteinized natural rubber by the number of citations (Table 1). This analysis showed research and review articles, for example, the document with the highest citation was a research article by researchers from France with 288 citations and 16.94 citations per year. This study consisted of developing corn starch nanocrystals and comparing the material with the behavior of natural rubber as a reinforcing agent in a thermoplastic starch matrix. The authors suggested that the nanocrystals presented two characteristics, the polarity of the material and the similarity between the chemical structures of the filler and the matrix. In such a way that starch nanocrystals lead to a slowing down of the recrystallization of the matrix during aging in the presence of moisture [27]. The document with the highest number of citations per year (26.42) was identified, which is possibly used for its significant contributions to the subject [28]. In addition, this was classified as the second document in a number of citations (185) and provides important results regarding the study of the genome of the *Hevea brasiliensis* tree. The research provided new insights into the physiology of laticifers and the molecular details of rubber biosynthesis, especially in relation to ethylene-stimulated rubber production. The tight control of ethylene synthesis under signaling and the active ethylene response in laticifers solves a long-standing mystery of ethylene stimulation in rubber production.

### 2.2. Cooccurrence-Centrality Analysis and Contingency Matrix

**Figure 2:**
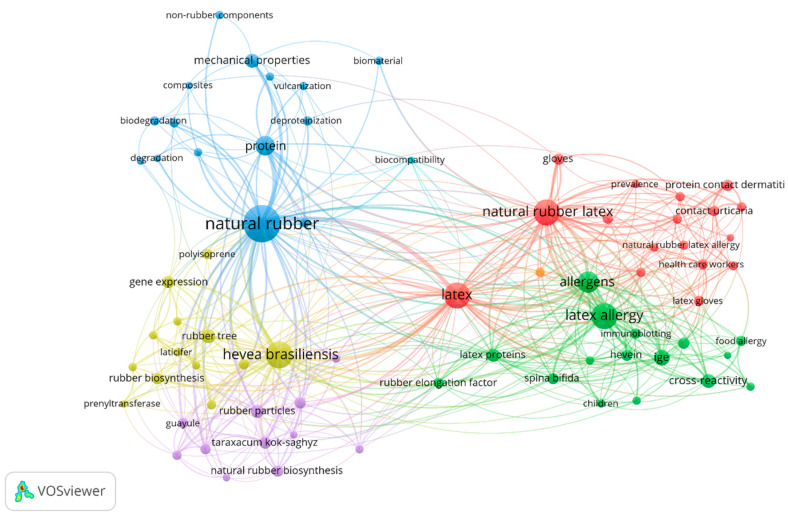
Bibliometric map of author keywords in publications on DPNR. Six theme groups: yellow, blue, green, purple, and red.

In order to map the most-used terms in the publications on DPNR, the bibliometric network was developed through a co-occurrence analysis. Figure 2 showed five groups of interrelated keywords with themes grouped in relation to the biosynthesis and obtaining of natural rubber from the species *Hevea brasiliensis*, guayule, and *Taraxacum kok-saghyz* (yellow and purple node). Terms associated with latex allergens and health complications (red and green nodes) were found. On the other hand, terms related to the effects of deproteinization on NR properties were identified, which include mechanical properties and degradation, among others (blue node). Additionally, the main reported keywords were determined based on their co-occurrence, being natural rubber (250), *Hevea brasiliensis* (104), natural rubber latex (100), latex (98), latex allergy (88), allergens (60), and protein (47)—the words used most closely in the documents published according to Scopus.

**Figure 3:**
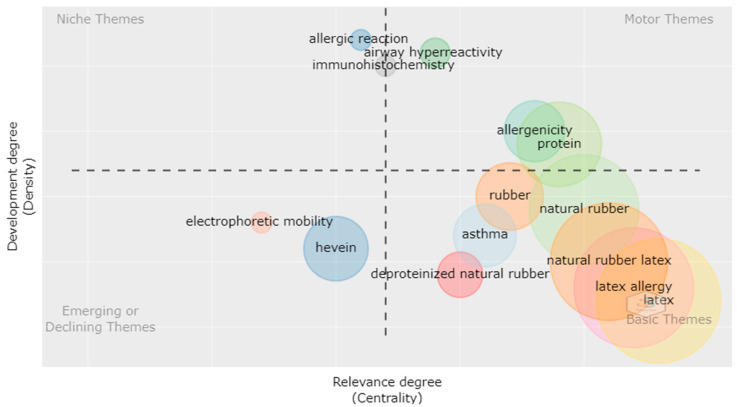
DPNR Author Keywords Strategy Chart (Bibliometrix).

Centrality and rank density are important tests for author keywords. Centrality is the degree of interaction of the topic of interest with other research topics, while density is the internal strength of the research topics [37]. Therefore, Figure 3 shows the centrality and density of terms through the strategic diagram of keywords in publications on deproteinized natural rubber. The map consisted of four quadrants with topic groups represented in circles. The larger the area of the circle, the higher the frequency of keywords in the group. The vertical axis represents keyword density and the horizontal axis represents centrality [38]. The first quadrant showed the themes that are developed and are important in the deproteinization of natural rubber (basic themes). The second and third quadrants allowed the identification of terms related to the allergenicity of natural rubber, which are relevant to the subject but are not yet fully developed (Motor themes and Niche themes). On the other hand, the fourth quadrant showed terms that are appearing and disappearing in research, that is, they are weakly developed topics such as electrophoretic mobility and what is related to the study of latex proteins by electrophoresis. On the other hand, the importance of this analysis is highlighted due to the identification of gaps and research opportunities that exist in the subject of natural rubber to be developed.

**Figure 4:**
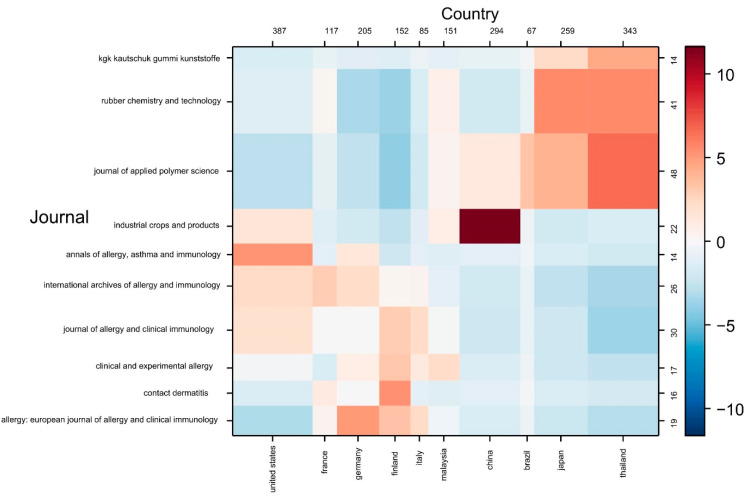
Contingency matrix on DPNR publications.

The CorText Manager scientometric platform was used to perform the contingency matrix and analyze the correlation between bibliometric parameters. This consisted of a map in which the colors indicate the degree of correlation between two variables under the measure of a statistical metric of chi-square co-occurrence. On the numerical scale, the value of −10 showed that the observed result of co-occurrence is 10 times lower than expected, on the other hand, the value of 10 indicated that the observed co-occurrence is 10 times higher than the expected value [39]. Additionally, the matrix presented a negative relationship through the blue cells, while the red cells meant a strong relationship; likewise, the white cells indicated that the variables had no relationship (neutrality) [40]. Figure 4 showed the correlations between the most relevant journals and countries in scientific production on NRPD. The analysis indicated that the United States has a correlation of 5 with the annals of allergy, asthma, and immunology (Q1–Q2), that is, there are 5 times more articles assigned between these factors than would be expected if the distributions of co-occurrence and semantic groups were independent [41]. Likewise, Thailand presented correlations higher than 5 with the Journal of Applied Polymer Science (Q2) and Rubber Chemistry and Technology (Q2–Q3), while China, for its part, showed a correlation of 10 with industrial crops and products (Q1). This analysis allowed us to infer the quality of the published studies and will help future researchers with the possible selection of journals in the respective fields of research.

### 2.3. Recent Advances in Deproteinization of NR

The removal of proteins from NR is one of the important topics in NR science and technology; therefore, the use of new methodologies that allow the removal of proteins is of interest for the development of materials from DPNR. For example, Chaikumpollert et al. [42] evaluated the implementation of organic solvents (ethanol, 2-propanol, and acetone) with urea and SDS surfactant for protein removal. The results showed that the combination of the agent’s urea, SDS, and the organic solvents presented the total removal of the proteins (0% *w/w* of Nitrogen) followed by two periods of centrifugation. Additionally, the authors determined the effect of deproteinization on the mechanical properties of NR, finding that the tensile strength of protein-free natural rubber (N% = 0.000% p/p) was significantly lower than that of natural rubber, although the tensile strength of DPNR (N% = 0.012% *w/w*) was similar to that of the parent rubber. Consequently, proteins and fatty acid ester groups were found to play an important role in the properties of natural rubber [18].

On the other hand, Tangboriboon et al. [43] reported the use of calcium chloride (CaCl_2_) and SDS for protein removal. CaCl_2_ was obtained by pyrolysis of egg shells, and it was found that the highest amount of soluble protein extracted (7338 µg/g) was achieved from SDS at 10% and 1.50 g of CaCl_2_. The effect of deproteinizing agents on the colloidal stability of NRFL has been analyzed. Abdullah et al. [44] reported that colloidal stability is governed by the SDS concentration so the increase in SDS concentration leads to an over-stabilization that can be detrimental to the properties of DPNR.

Protein removal is a process that can influence other types of properties such as the degradation of natural rubber, which can be influenced by the proteins present in the rubber since proteins are capable of adsorbing water and oxygen [45]. Yamamoto et al. [46] evaluated the thermal degradation of high ammonia NR and DPNR. The authors reported that the degradation for NR was of the oxidative type, while the degradation of DPRN was non-oxidative, that is, NR had the nanomatrix structure of the non-rubber components so that the water and oxygen molecules adsorbed by the proteins enabled oxidative degradation. Furthermore, DPNR has been reported to be used to develop bioproducts such as face masks, transdermal nicotine patches, and drug delivery vehicles [47,48]. For example, Pichayakorn et al. [49], formulated a removable thin film from DPNR of *Hevea brasiliensis*. The in-situ film was of good elasticity indicated by the initial modulus (0.96 MPa), tensile strength (0.208 MPa), and elongation at break (105.6%). The skin irritation on the rabbit skin was very mild, which showed a little skin rash, but with quick recovery. The compliance test on 50 healthy volunteers showed good results with no irritation effect. In conclusion, DPNR could be a ready-made peel-off mascara product with good properties.

The information described in the bibliometric analysis made it possible to describe the panorama of DPNR and its importance. This allowed us to identify the need to implement processes that can be adapted to an industrial scale to obtain DPNR clots, therefore, in the present work, homogenization conditions of deproteinizing agents and the effect of deproteinization on coagulation were evaluated on NR. It is noteworthy that the reports on NRFL protein removal focus on laboratory-scale methodologies for the removal of allergens and the characterization of their properties.

## 3. Materials and Methods

### 3.1. Materials and Reagents

The NRFL was obtained from the Cauca River zone, Antioquia-Colombia. The samples stabilized with ammonium laurate (0.01%) were supplied by the RubberCorp latex concentrator company (Caucasia, Colombia). The suitable storage condition of NRFL was at room temperature 30 °C in tight containers where NRFL could be kept for longer than 4 months. The deproteinization agents used were commercial grade purchased from Protokimica Ltd., Medellin, Colombia: sodium dodecyl sulfate (SDS), Urea (U), Tween 80 (T80), Ethanol (96%) (-OH), polyethylene glycol 400–6000 (PEG400 and PEG6000), and acetic acid. Folin-Ciocalteu reagents (Sigma-Aldrich, Burlington, MA, USA) and copper (II) tartrate hydrate (Merck) were used in the Lowry method.

### 3.2. NRFL Deammonification Methods

Latex samples were deammonized in triplicate by three deammonification methods, Vortex, Sonication, and Cowles at 70 RPM for 24 h, followed by alkalinity quantification, down to an approximate value of 0.1%.

### 3.3. Preparation of DPNR Samples

The NRFL samples were prepared in 50 mL Falcon tubes and the deproteinization agents were added, which were mixed under vortex agitation for 30 s [50,51]. Subsequently, the samples were left in incubation for 24 h and protein measurements were made. Table 2 shows the formulations of the deproteinizers and their concentrations.

### 3.4. Protein Measure

Total protein quantification in NRFL was performed using the Kjeldahl method (ISO 1871:2009) [52,53]. A portion of deproteinized NRFL is subjected to pretreatment by adding phosphate buffer (pH 8) volume ratio 1:3 and centrifugation at 9500 RPM for 40 min. Then, the solid located in the upper part of the tube is discarded and the aqueous phase is centrifuged again at 14,000 RPM for 20 min [54]. Water-soluble proteins were quantified by the modified Lowry method [55].

The percentage of protein removal from the NR (%PR) was determined using Equation (1), where L is the percentage of proteins in the aqueous phase after pretreatment and K is the total protein content in NRFL. It is noteworthy that the nitrogen content provided by ammonium laurate and urea was initially subtracted in the calculations. In addition, three methods of mixing the deproteinizing agents were evaluated: Vortex, Sonication, and Cowles method. Statistical analysis of the results was performed by analysis of variance (ANOVA) with 95% reliability, using the statistical software STATGRAPHICS Centurion version XVI.

(1) $$\%PR=\frac{L}{K}\times 100\;$$

### 3.5. Zeta Potential Analysis

Zeta potential analysis was determined using a Litesizer 500 Anton Paar particle analyzer (Graz, Austria). About 5–10% (*v/v*) of rubber latex was dispersed in distilled water before the analysis Zeta potential was observed using the conversion of the frequency and scattering intensity function [56].

### 3.6. Effect of Concentration on the Deproteinized Systems over Protein Removal

The systems that presented the best protein removal were analyzed using a single-factor experimental design with six levels (Table 3). Urea and ethanol concentrations remained constant at 0.1 and 0.025%, respectively.

### 3.7. DPNR Coagulation

NR and deproteinized latex samples were diluted 50% in water and coagulated with 10% acetic acid at 10% concentration, then the mixture was quickly stirred and left to stand until coagulation.

### 3.8. Plasticity

Plasticity measurements were performed on samples deproteinized with SDS, SDS + U and SDS + U + OH. These DPNR were obtained by Cowles for homogenization of the deproteinizing agents and 2% concentrations were used. Plastometer H-01 CGM technology (CG Engineering Company Ltd., Pathum Thani, Thailand) was used to measure the plasticity (P_0_) and plasticity retention index (PRI) of the obtained DPNR.

## 4. Results

### 4.1. NRFL Deammonification Methods

**Figure 5:**
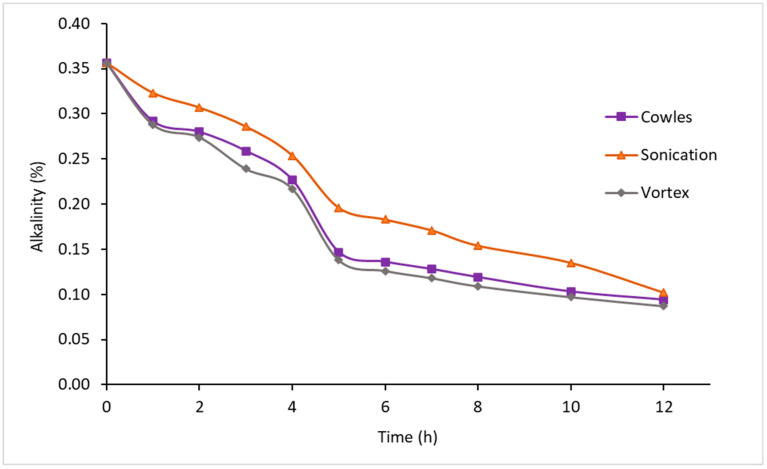
NRLF deammonification.

The deammonification of NR was carried out with the purpose of reducing the concentration of ammonia, which can affect the coagulation of NR due to the over-stabilization generated by high concentrations of ammonia (˃0.60% alkalinity) [57]. Figure 5 showed that the three methods evaluated reached the approximate value of 0.10% alkalinity at 12 h of treatment. It is noteworthy that the Vortex and Cowles methods presented alkalinity values below the established limit with 0.087 and 0.094%, respectively. So that the spiral rotational flow of the vortex and the high shear levels transmitted by the Cowles helices possibly increase the ammonia volatilization flux due to increased air-liquid contact. It was also identified that the non-deammonified NRFL corresponds to a low ammonia latex (around 0.30% alkalinity). Additionally, the results showed that a method with industrial projection such as Cowles can be used to reduce the ammonia levels and promote coagulation of the NR, since the Vortex method may be limited for use on a larger scale.

### 4.2. Deproteinization Systems

**Figure 6:**
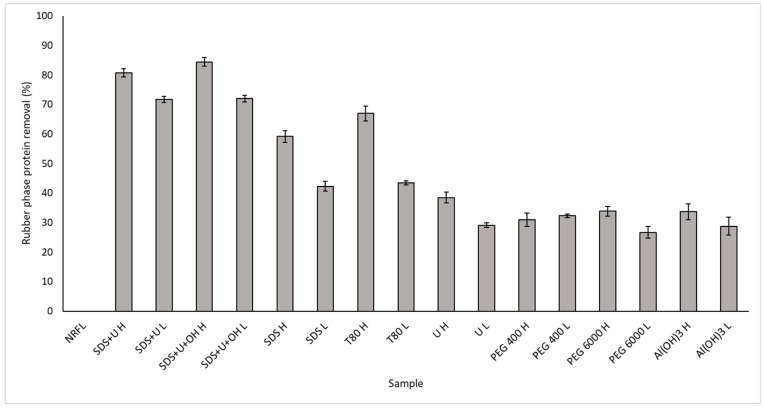
Deproteination systems.

The removal of NRFL protein from the lower basin of the Cauca River was analyzed by chemical deproteinization methods. Figure 6 shows that the systems with SDS and Tween 80 formulations present removals of 42.3–84.4% and 43.4–67.0%, respectively. This can be attributed to the ability of SDS to denature proteins through its effect on non-covalent bonds [43]. SDS anions bind to the main peptide chains in a ratio of one SDS anion for every two amino acid residues, which adds a negative charge to the protein. Therefore, SDS binding enables electrostatic repulsion that allows proteins to unfold into an α-helix [57]. In addition, SDS has been reported to be one of the most effective surfactants for improving NRFL stability due to the ease of modifying the latex surface [58]. On the other hand, Tween 80 can favor protein solubility and retain protein–protein interactions, in such a way that this effect affects the removal of proteins in the NRFL.

On the other hand, urea is known to affect the spatial conformation of proteins [19]. Furthermore, proteins tend to bind on the surface of urea particles through physical interactions [18]. For this reason, the systems containing urea such as SDS + U and SDS + U + OH presented removals higher than 70%. This suggests that the addition of Urea potentiated the ability to remove proteins in NRFL. Additionally, the effect of an organic solvent on protein removal was evaluated, finding the highest removal value of 84.4% in the SDS + U + OH H treatment. This result is possibly due to the effect of the dielectric constant of ethanol, leading to decreased protein solubility [18]. Chaikumpollert [42] evaluated different solvents (ketone, ethanol, and 2-propanol) in the deproteinization of NR and found a 100% removal when 0.025% ethanol was used in the presence of urea and SDS, consequently, the use of ethanol is suggested to obtain DPNR from NRFL of the lower basin of the Cauca River. The result DPNR was white liquid, like milk, that was not much different from fresh NRFL. The pH value of DPNR was 10.08 which was higher than that of fresh NRFL 9.67. It is highlighted that the NRFL systems were deammonified with the Cowles method to a 0.094% alkalinity for the rest of the experiments.

### 4.3. Zeta Potential

**Figure 7:**
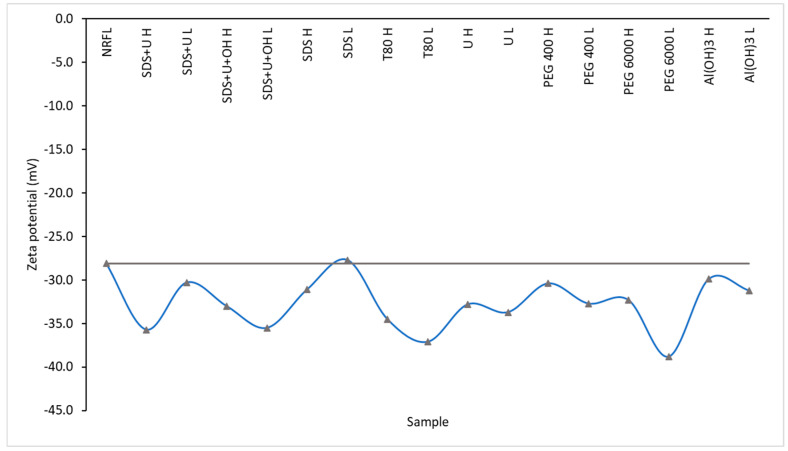
Zeta potentials of deproteinized systems.

The NRFL usually maintained colloidal stability via their surface charges having either lower than −30 mV or higher than +30 mV [56]. Zeta potentials of NRFL and DPNR showed negative values, indicating the presence of negative charges on the surfaces of the rubbers (Figure 7). It was found that all rubber samples were a negative charge at pH 9.67–10.08. The zeta potential values of DPNR latex were lower than NFRL except for low SDS (−27.7 mV). This may be due to the SDS concentration being too low. Therefore, after the removal of protein, the negative charge of rubber particles was reduced but still remained stable without self-coagulation. On the other hand, Ariyawiriyanan [56] suggests that the zeta potential has an incidence on the particle sizes of the DPNR, finding that at lower zeta potentials the particle size decreases. Therefore, it is inferred that possibly the smallest particle sizes were obtained from the system PEG6000 L. The zeta potential of Colombian NRFL shows similar values as reports of other countries [44,59,60].

### 4.4. Effect of Concentration of the Deproteinized Systems over Protein Removal

**Figure 8:**
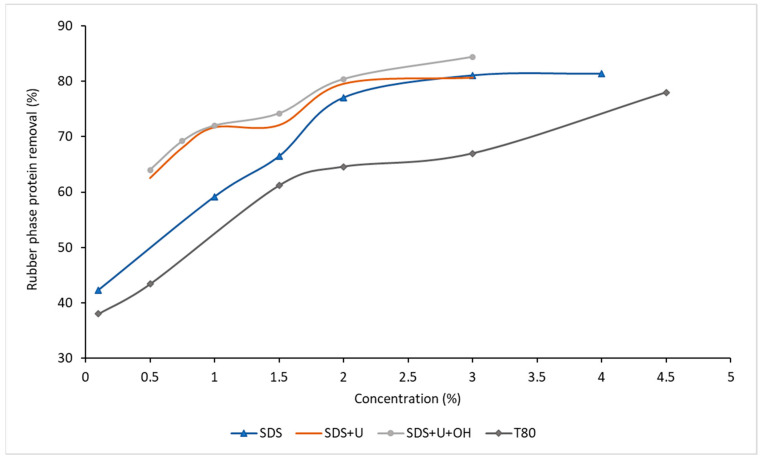
Effect of concentration on the deproteinized systems over protein removal.

In Figure 8, it is observed that the systems composed of SDS present greater protein removal at higher concentrations, and the removal increases with the addition of urea and, in turn, increases with the addition of ethanol for all concentrations used. This can be explained by the migration of proteins to a system where the organic solvent with a high dielectric constant allows greater protein removal.

### 4.5. Effect of Homogenization Methods on Protein Removal

**Figure 9:**
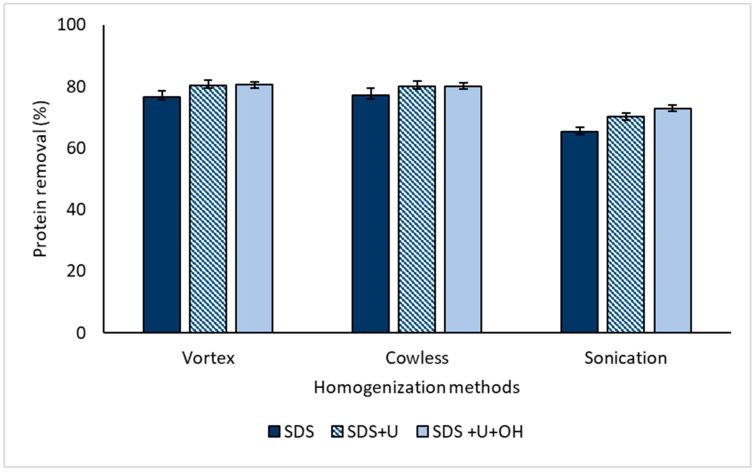
Effect of homogenization methods on protein removal (2% concentration of deproteinizing agents).

Figure 9 shows the protein-removal values for the different deproteinizer homogenization methods, finding that the Vortex and Cowles methods presented the highest removal percentages from the SDS + U and SDS + U + OH systems with values of 80.41–80.50% and 80.10–80.22%, respectively. Therefore, it is highlighted that the homogenization of the deproteinizing agent can be carried out from Cowles without negatively affecting protein removal, which indicates that this system can possibly be implemented on an industrial scale to obtain NR reduced by 80% of proteins. The results of removal by Vortex and Cowles did not present significant differences (*p*-value ˃ 0.95).

### 4.6. DPNR Coagulation

**Figure 10:**
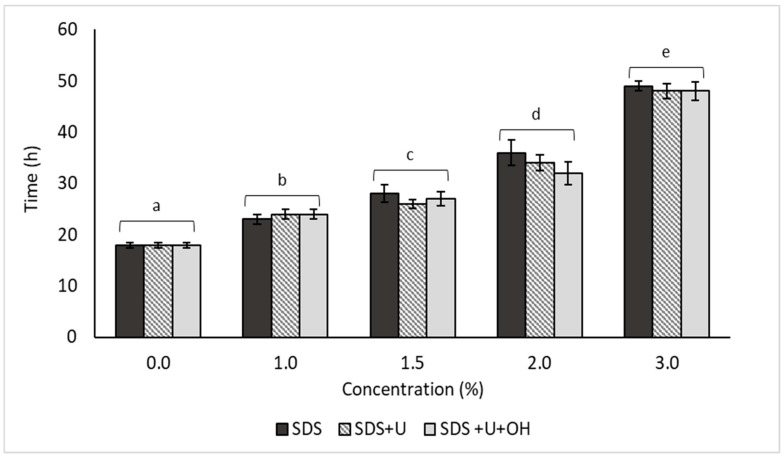
Coagulation of protein-reduced systems, equal letters correspond to statistically homogeneous groups using the LSD multiple range test.

The systems with higher percentages of protein removal were subjected to coagulation in order to obtain DPRN. Figure 10 showed that the addition of deproteinizing agents tends to over-stabilize the field latex. The results showed significant differences between treatments under the different concentrations (*p*-value < 0.95). Therefore, the concentration of the deproteinizing agent affects the coagulation time of DPRN. For example, it was found that the 3% concentration presented the longest coagulation times with values of 49, 48, and 48 h for SDS, SDS + U and SDS + U + OH, respectively. Consequently, increasing the concentration of the deproteinizing agent increases coagulation times. On the other hand, it was identified that the 2% concentration can be considered to obtain NR clots with 80% protein removal in 32, 34, and 36 h for SDS, SDS + U, and SDS + U + OH, respectively. It is noteworthy that in this work a centrifuge was not used to separate the denatured proteins soluble in the aqueous phase; therefore, the coagulation of the NR allowed the separation of the proteins.

### 4.7. Plasticity

The Plasticity Retention Index (PRI) has been developed as an objective test for the oxidizability of raw natural rubber and has been associated with the Standard Malaysian Rubber Scheme. Oxidizability, as measured by PRI, significantly affects behavior of rubber in the factory and in-service performance [61]. Table 4 shows the PRI values for the protein-reduced rubbers. The results indicated that the addition of SDS, urea, and ethanol decreased the PRI. In addition, the NR of the lower Cauca river basin, Antioquia-Colombia was found to have a high PRI value of 93.3% and was higher than that reported by Osabohien [62] for NR Standard Nigerian Rubber 10 with a value of 71.0%. It should be noted, the high values of the plasticity retention index indicate a high resistance of the rubber to aging.

## 5. Conclusions

Scientometrics is characterized by offering the possibility of extracting patterns from large databases, in order to provide evidence of the impact of research results. Thus, this bibliometric analysis revealed trends in technological advances related to the biosynthesis and production of NR, NRFL allergens, and the effects of deproteinization on NR properties, which include mechanical properties and degradation, among others. In addition, the bibliometrics showed the evolution of research in DPNR, finding significant growth in the United States, Thailand, and Germany. This provides an encouraging outlook in the search for alternatives for NRFL deproteinization, which may favor the use of DPNR in markets that are currently unexplored.

On the other hand, the evaluation of the deproteinization of NFRL allowed us to find for eight deproteinization systems that the presence of SDS and Tween 80 surfactants affected protein removal in NRFL. It was also possible to observe that the addition of Urea in the formulations with SDS increased the protein removal, and in turn, all the removal percentages were exceeded with the addition of Ethanol in the systems. On the other hand, all deproteinizing systems decreased NRFL Zeta potentials without self-coagulation, suggesting enhanced colloidal stability in DPNR latex.

It was possible to observe the implementation of a Cowles system as a method of deammonification and homogenization of the deproteinizing agent, which can possibly be used on an industrial scale to obtain DPRN. Additionally, it was found that increasing the concentration of the deproteinizing agent increased the clotting times of the DPNR. On the other hand, Plasticity measurements indicated that the addition of SDS, urea, and ethanol decreased the %PRI in the protein-reduced rubbers.

## Figures and Tables

**Table 1:** Documents with the highest citations in global research on DPNR.

Title	Journals	Authors Affiliation Countries	Number of Citations	Number of Citations per Year	References
Thermoplastic starch—Waxy maize starch nanocrystals nanocomposites	*Biomacromolecules*	France	288	16.94	[27]
The rubber tree genome reveals new insights into rubber production and species adaptation	*Nature plants*	China	185	26.42	[28]
Structural Characterization of Natural Polyisoprenes: Solve the Mystery of Natural Rubber Based on Structural Study	*Rubber Chemistry and Technology*	Thailand	176	8.00	[29]
Draft genome sequence of the rubber tree *Hevea brasiliensis*	*BMC Genomics*	Malaysia	168	16.80	[30]
Sensitization to cross-reactive carbohydrate determinants and the ubiquitous protein profilin: mimickers of allergy	*Clinical and Experimental Allergy*	Belgium	160	8.42	[31]
Primary prevention of natural rubber latex allergy in the German health care system through education and intervention	*Journal of Allergy and Clinical Immunology*	Germany	158	7.52	[32]
Surface nanostructure of *Hevea brasiliensis* natural rubber latex particles	*Colloids and Surfaces A: Physicochemical and Engineering Aspects*	Thailand, Malaysia, The Netherlands	149	12.41	[33]
Cornstarch powder on latex products is an allergen carrier	*Journal of Allergy and Clinical Immunology*	United States	145	5.00	[34]
Transcriptome analysis reveals novel features of the molecular events occurring in the laticifers of *Hevea brasiliensis* (para rubber tree)	*Plant Molecular Biology*	Malaysia	144	7.20	[35]
Rubber contact urticaria	*Contact Dermatitis*	Finland	142	4.05	[36]

**Table 2:** Deproteinization systems.

Component	Abbreviation	Concentration (%)
Sodium dodecyl sulfate	SDS L	0.1
	SDS H	1.0
Sodium dodecyl sulfate y Urea	SDS + U L	1.0 + 0.1
	SDS + U H	3.0 + 0.1
Sodium dodecyl sulfate, Urea y Ethanol	SDS + U + OH L	1.0 + 0.1 + 0.025
	SDS + U + OH H	3.0 + 0.1 + 0.025
Tween 80	T80 L	0.5
	T80 H	3.0
Urea	U L	0.8
	U H	1.0
Polyethylene glycol 400	PEG400 L	0.4
	PEG400 H	0.8
Polyethylene glycol 6000	PEG6000 L	0.07
	PEG6000 H	0.15
Aluminum Hydroxide	Al(OH)_3_ L	0.15
	Al(OH)_3_ H	0.30

**Table 3:** Design of experiments to improve protein removal.

Component	Factor	Concentration (%)
Sodium dodecyl sulfate	SDS	0.1; 1.0; 1.5; 2.0; 3.0 y 4.0
Sodium dodecyl sulfate and Urea	SDS + U	0.5; 0.75; 1.0; 1.5; 2.0 y 3.0
Sodium dodecyl sulfate, Urea, and Ethanol	SDS + U + OH	0.5; 0.75; 1.0; 1.5; 2.0 y 3.0
Tween 80	T80	0.1; 0.5; 1.5; 2.0; 3.0 y 4.5

**Table 4:** Initial plasticity and plasticity retention index of DPNR Colombian from *Hevea brasiliensis*.

Treatment	Protein Removal (%)	Initial Plasticity (P_0_)	Plasticity Retention Index (%PRI)
SDS	77.1	36.8	86.4
SDS + U	80.2	38.2	84.2
SDS + U + OH	80.1	37.4	83.2
Non deproteinized NR	NA	34.8	93.3
NR Standard Nigerian Rubber 10 [62]	NA	NA	71.0
Saponification, SDS and proteolytic enzyme [63]	78.4, 96.9, and 81.5	NA	10–30
Urea with three cycles of centrifugation [64]	82.0	70.0	80.0
SDS, Urea [65]	NA	54.0	27.0

NA: not applicable.

## Data Availability

Not applicable.

## Sample Availability

Samples of the compounds are not available from the authors.
